# Is There Still a Role for Two-Phase Contrast-Enhanced CT and Virtual Monoenergetic Images in the Era of Photon-Counting Detector CT?

**DOI:** 10.3390/diagnostics13081454

**Published:** 2023-04-18

**Authors:** Arne Estler, Konstantin Nikolaou, Stefan O. Schönberg, Fabian Bamberg, Matthias F. Froelich, Fabian Tollens, Niklas Verloh, Jakob Weiss, Marius Horger, Florian Hagen

**Affiliations:** 1 Department of Diagnostic and Interventional Radiology, University of Tuebingen, Hoppe-Seyler-Str. 3, 72076 Tuebingen, Germany; 2 Department of Radiology and Nuclear Medicine, University Medical Centre Mannheim, Medical Faculty Mannheim, University of Heidelberg, 68167 Mannheim, Germany; 3 Department of Diagnostic and Interventional Radiology, Medical Center University of Freiburg, 79106 Freiburg, Germany

**Keywords:** photon counting CT, dual-source CT, dual-phase contrast-enhanced CT

## Abstract

Background: To compare the diagnostic characteristics between arterial phase imaging versus portal venous phase imaging, applying polychromatic T3D images and low keV virtual monochromatic images using a 1st generation photon-counting CT detector, of CT in patients with hepatocellular carcinoma (HCC). Methods: Consecutive patients with HCC, with a clinical indication for CT imaging, were prospectively enrolled. Virtual monoenergetic images (VMI) were reconstructed at 40 to 70 keV for the PCD-CT. Two independent, blinded radiologists counted all hepatic lesions and quantified their size. The lesion-to-background ratio was quantified for both phases. SNR and CNR were determined for T3D and low VMI images; non-parametric statistics were used. Results: Among 49 oncologic patients (mean age 66.9 ± 11.2 years, eight females), HCC was detected in both arterial and portal venous scans. The signal-to-noise ratio, the CNR liver-to-muscle, the CNR tumor-to-liver, and CNR tumor-to-muscle were 6.58 ± 2.86, 1.40 ± 0.42, 1.13 ± 0.49, and 1.53 ± 0.76 in the arterial phase and 5.93 ± 2.97, 1.73 ± 0.38, 0.79 ± 0.30, and 1.36 ± 0.60 in the portal venous phase with PCD-CT, respectively. There was no significant difference in SNR between the arterial and portal venous phases, including between “T3D” and low keV images (*p* > 0.05). CNR_tumor-to-liver_ differed significantly between arterial and portal venous contrast phases (*p* < 0.005) for both “T3D” and all reconstructed keV levels. CNR_liver-to-muscle_ and CNR_tumor-to-muscle_ did not differ in either the arterial or portal venous contrast phases. CNR_tumor-to-liver_ increased in the arterial contrast phase with lower keV in addition to SD. In the portal venous contrast phase, CNR_tumor-to-liver_ decreased with lower keV; whereas, CNR_tumor-to-muscle_ increased with lower keV in both arterial and portal venous contrast phases. CTDI and DLP mean values for the arterial upper abdomen phase were 9.03 ± 3.59 and 275 ± 133, respectively. CTDI and DLP mean values for the abdominal portal venous phase were 8.75 ± 2.99 and 448 ± 157 with PCD-CT, respectively. No statistically significant differences were found concerning the inter-reader agreement for any of the (calculated) keV levels in either the arterial or portal-venous contrast phases. Conclusions: The arterial contrast phase imaging provides higher lesion-to-background ratios of HCC lesions using a PCD-CT; especially, at 40 keV. However, the difference was not subjectively perceived as significant.

## 1. Introduction

Dual-phase or even three-phase contrast-enhanced liver CT examination protocols have already been recommended decades ago in order to more accurately detect primary or secondary lesions—smaller, hyper-vascularized in particular—otherwise expected to be missed in the portal venous contrast phase (pv-phase); the latter, being the most frequently used contrast phase in abdominal imaging (“FDA Public Health Notification: Reducing Radiation Risk from Computed Tomography for Pediatric and Small Adult Patients,” 2002; Huda et al., 2000; Kalra et al., 2004; McCollough et al., 2006; Papadakis et al., 2007) [1,2,3,4,5]. This knowledge still has its plausibility, but it was propagated mainly in earlier times while using less performant CT scanners (slower table speed, longer reconstruction times, longer detector cooling time, etc.). Nevertheless, some malignant tumors such as hepatocellular carcinomas, neuroendocrine tumors, sarcomas, and even some of the most frequent malignancies such as breast, lung or GI, and GU malignant neoplasm may enhance early so that the contrast blush fades away seconds after hampering lesion detection if only pv-phases are used (Higashigaito et al., 2022; Hsieh et al., 2021; Rajendran et al., 2022; Tabatabaei et al., 2020; Wichmann et al., 2017) [6,7,8,9,10]. In the last ten years, with the advent of dual-energy CT detectors, lesion detection was generally improved by using virtual monoenergetic images with lower keV (virtual mono-contrast) images (Shuman et al., 2014a; Yamada et al., 2014) [11,12]. Besides, known physiological differences in liver and liver lesion perfusion are still expected; which, depend on the blood supply to both tissues (via the hepatic artery or the portal vein, or both), their microarchitecture, size of the extravascular, interstitial space, tumor flow characteristics and such that are protocol-dependent (contrast agent volume, contrast medium concentration, flow, volume of saline chaser, circulation time, etc.), differences in the attenuation, and delineation between the lesion and the liver parenchyma. However, they have to be enhanced by improving tissue contrast. In addition to liver lesion imaging, multiple dual-energy—and virtual monoenergetic image reconstruction—applications exist in the literature. Examples of applications here include imaging of the coronary arteries, detection of intra-abdominal hemorrhage, and CT angiography of the carotid and intracerebral vessels [13,14,15,16,17,18].

With the advent of the photon-counting detector CT, each individual X-ray that passes through the patient’s body is directly converted by incoming photons into electronic signals proportional to their deposited energy being immune to electronic noise [19,20,21,22,23]. The count rate is not affected by electronic noise. These properties have the potential for more accurate lesion delineation and increased tissue contrast. Moreover, using low keV for the generation of virtual mono-contrast images, enhancement characteristics (peripheral vs. central, diffuse vs. focal, heterogeneity of microvasculature) are expected to be further enhanced improving the detection of focal hepatic lesions.

Encouraged by these assumptions, we initiated a prospective comparative (arterial vs. portal venous) study in patients with follow-up for hepatocellular carcinomas, with the intention to compare the sensitivity of portal venous monophasic polychromatic T3D PCD-CT images with those of a combined arterial and portal venous dual-phase contrast-enhanced PCD-CT examinational protocol for the detection of hepatocellular carcinomas in the liver, and additionally, to assess the eventual benefit of complementary virtual mono-contrast 40–70 KeV reformates over the primarily generated T3D images. For this purpose, we evaluated differences in terms of SNR, CNR, as well as an inter-reader agreement for all images generated.

## 2. Materials and Methods

### 2.1. Subjects

Our institutional review board approved this prospective data evaluation, which was assigned the approval number 696/2021B01. Participants gave written informed consent. Between October 2021 and July 2022, a total of 49 consecutive patients with known hepatocellular carcinoma, who were referred for staging or treatment monitoring to our radiology department and who had standardized previous CT exams or MRI exams to confirm the HCC suspicious lesions at our institution, were enrolled.

### 2.2. CT-Examinational Protocol on PCD-CT

*Arterial phase: scans were all acquired on a 1st generation photon-counting detector CT with quantum imaging (NAEOTOM Alpha, Siemens Healthineers, Forchheim, Germany) equipped with two photon-counting detectors. The following examinational protocol was used: 120 KvP, eff. mAs 144, quantum iterative reconstruction factor 2 (QIR 2), level 145, slice thickness 3 mm, focal spot 0.8/1.2, kernel Br40f, single collimation width 0.4 mm, total collimation width 57.6 mm, table speed 92 cm/s, table feed/rotation 46, and spiral pitch factor 0.8.

*Portal venous phase: 120 KvP, eff. mAs 148, quantum iterative reconstruction factor 2 (QIR 2), level 145, slice thickness 3 mm, focal spot 0.8/1.2, kernel Br40f, single collimation width 0.4 mm, total collimation width 57.6 mm, table speed 92 cm/s, table feed/rotation 46, and spiral pitch factor 0.8.

### 2.3. Contrast Agent Protocol

The contrast medium protocol included intravenous administration of 1.2 mL/kg/BW (IMERON 350 mg iodine/mL [BRACCO Imaging, Konstanz, Germany]) at a flow rate of 2 mL/s via antecubital vein followed by a saline flush of 50 mL NaCl at 2.5 mL/s for both phases and in all patients. Contrast material was administered by using a dual-head pump injector (CT motion XD 8000, Ulrich Medical, Ulm, Germany). The delay between contrast agent injection and the scan was 30 s for the arterial phase and 65 s for the portal venous phase for PCD-CT.

### 2.4. Image Reconstructions

**Figure 1:**
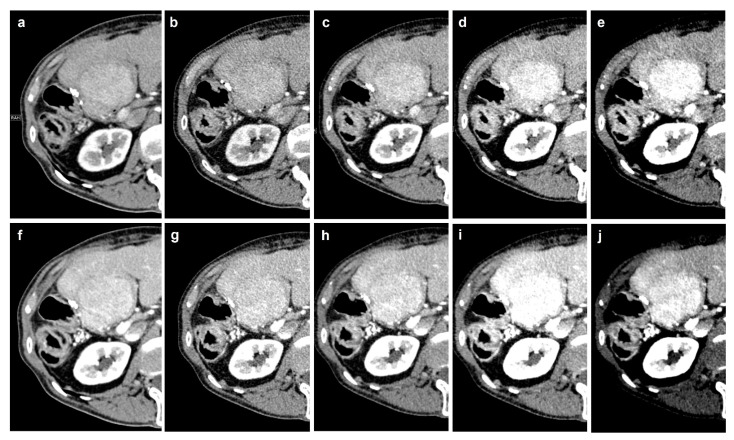
Example of the different reconstructions in an HCC lesion: (**a**–**e**): arterial phase imaging (T3D, 70 keV, 60 keV, 50 keV, 40 keV); (**f**–**j**) portal venous imaging (T3D, 70 keV, 60 keV, 50 keV, 40 keV).

PCD-CT was performed at 120 kVp in QuantumPlus mode (obtaining full spectral information) and polychromatic T3D images were generated. Slice thickness was 3 mm for both series; single slice collimation was 0.4 mm. The kernel was set at Br40f for both phases with quantum iterative reconstruction factor 2 (QIR 2). The spiral pitch factor was 0.8. Virtual monoenergetic images (VMI) were reconstructed at 40 to 70 keV for the PCD-CT (Figure 1).

### 2.5. Subjective Image Analysis

VMI arterial and portal venous abdominal CT images were read in consensus by two radiologists with 5 and 4 years of experience in abdominal imaging. Images were randomly analyzed (arterial and portal venous, timely apart from each other) with freely adjustable window settings. Subjective image contrast and image noise were evaluated by using a five-point Likert scale: 1, excellent image quality; 2, good image quality; 3, fair but comprised image quality; 4, poor image quality; 5, non-diagnostic. Disagreements were resolved during a final consensus round.

### 2.6. Objective Image Analysis

All lesions were counted and registered. The maximum diameter was measured. In each patient, round or oval ROIs were manually placed twice within the liver (ROI size, 100–200 mm^2^), once within the lesion (40–100 mm^2^), once within the portal vein (30–60 mm^2^), and twice within the psoas muscle (40–100 mm^2^) depending on their size.

### 2.7. Quantitative Evaluation of Image Quality and Noise

In every patient, one index lesion was defined and considered for the following calculations. Signal-to-noise ratios (SNR) of the liver lesions were calculated as follows: SNR = (HUROI)/SDROI_liver_. The contrast-to-noise ratio (CNR) for the liver-to-muscle, tumor-to-liver, and tumor-to-muscle ratios was calculated as follows: CNR_liver-to-muscle_ = (mean HU of ROI_liver_/mean HU of ROI_muscle_); CNR_tumor-to-liver_ = (mean HU of ROI_lesion_/mean HU of ROI_liver_); CNR_tumor-to-muscle_ = (mean HU of ROI_lesion_/mean HU of ROI_muscle_).

Image noise was defined as the standard deviation (SD) of the psoas muscle (SD_muscle_). All ratios were performed between 40 and 70 keV at the arterial and portal venous phases in PCD-CT and for the T3D images at the arterial and portal venous phases.

### 2.8. Radiation Metrics

In all patients, the volumetric CT dose index (CTDIvol) and dose-length product (DLP) were documented from the dose report; which, was automatically stored in the picture archiving and communication system. Subsequently, the absolute values were compared between both contrast phases.

### 2.9. Statistical Analysis

Data analysis was performed using IBM SPSS Statistics for Windows, Version 26.0 (IBM Corp., Armonk, NY, USA). The level of significance was set at α = 0.05. Continuous variables are provided as mean ± standard deviation (95% confidence interval). Normal data distribution was assessed by applying the Shapiro–Wilk test. In the case of normal distribution, the variables of the two groups were compared according to the t-test for pairs. The Wilcoxon signed-rank paired test was used if data were not normally distributed. Comparison between the different keV levels of the PCD-CT was compared with the Friedman test, followed by post hoc Dunn–Bonferroni tests with alpha correction to analyze differences between the subgroups, if necessary.

## 3. Results

### 3.1. Patient Characteristics

A total of 49 patients (41 male, 66.9 ± 11.2 years) were included. Of them, 94% (*n* = 46) had a previous MRI for validation of the liver lesions with a mean time difference of 4.6 months (SD ± 3.4 months) (Table 1). The remaining three had only a multiphase dual-source CT (DSCT).

### 3.2. Clinical Evaluation

Overall, between one and >10 hepatic HCC lesions were found in patients in the current study using PCD-CT. There was no statistically significant difference between arterial and portal venous contrast phases in detecting tumor manifestations (see Table 2); none of the arterialized liver lesions were missed in the portal venous contrast phase. The arterial contrast phase showed no clinical benefit in terms of the detectability of hepatocellular carcinoma manifestations.

### 3.3. Arterial vs. Portal Venous Contrast Phase

In terms of objective image quality, we found that the “liver-to-muscle” contrast-to-noise ratio and the “tumor-to-liver” CNR differed significantly between arterial and portal venous contrast phases in PCD-CT (*p* < 0.005). The signal-to-noise and contrast-to-noise ratios of “tumor-to-muscle” did not differ significantly (*p* = 0.416 and 0.366, respectively, see Table 3).

#### Evaluation of Calculated Low keV Images

**Figure 2:**
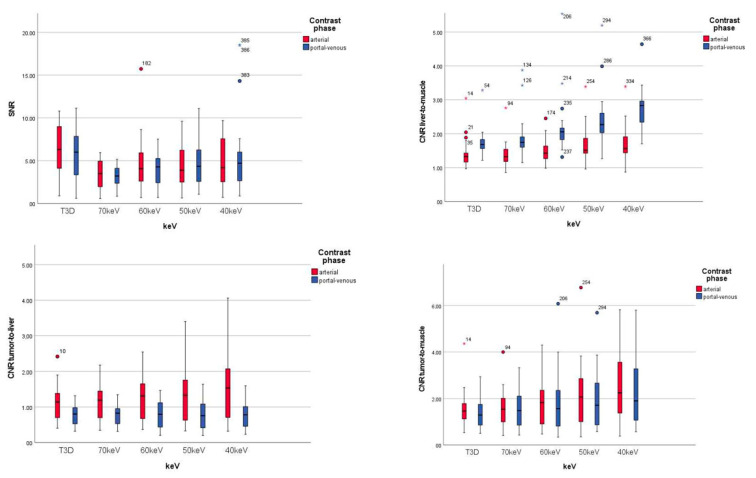
Comparison of different keV levels in terms of SNR and CNR.

In terms of the signal-to-noise ratio, there was no statistically significant difference between “T3D” and the respective (low) keV levels; neither for arterial nor portal venous (*p* > 0.05, see Figure 2 and Table 4). In addition, the “liver-to-muscle” contrast-to-noise ratio, the “tumor-to-liver” CNR, and the “tumor-to-muscle” CNR did not differ with regard to SNR in either the arterial or portal venous contrast phases. The “liver-to-muscle” CNR increased in both arterial and portal venous contrast phases; whereas, the keV decreased while the standard deviation increased. There were statistically significant differences in terms of the CNR_tumor-to-liver_ when comparing arterial to portal venous phases through all reconstructions (T3D to 40 keV, see Table 5). Image noise, which was determined using the standard deviation of the psoas muscle, did not differ between arterial and portal venous contrast phases (Table 6).

The “tumor-to-liver” CNR increased in the arterial contrast phase with a lower keV whereas, the standard deviation increased sharply. With respect to the portal venous contrast phase, “tumor-to-liver” CNR decreased when the keV value was decreased.

The “tumor-to-muscle” CNR increased when the keV value was reduced for both arterial and portal venous contrast phases.

The Friedmann test revealed a value of <0.001 with regard to the SNR of both the arterial and portal venous reconstructions; thus, the central tendencies differ with regard to SNR. We found a statistically significant difference between T3D and 70 keV after Bonferroni alpha correction in both arterial and portal venous contrast phases (Table 7 and Table 8).

In terms of the arterial contrast phase CNR_liver-to-muscle,_ we only found a statistically significant difference in the comparison of T3D and 40 keV after Bonferroni alpha correction (Table 9).

In terms of the portal venous contrast phase CNR_liver-to-muscle,_ we found statistically significant differences in the comparison of T3D and 60/50/40 keV as well as 70 keV and 50/40 keV after Bonferroni alpha correction (Table 10).

We did not find any statistically significant differences in terms of arterial or portal-venous CNR_tumor-to-liver_ (Table 11 and Table 12).

Additionally, we did not find any statistically significant differences in terms of arterial CNR_tumor-to-muscle_ (Table 13).

After Bonferroni alpha correction, we found statistically significant differences comparing T3D and 50/40 keV as well as comparing 70 keV with 40 keV (Table 14).

### 3.4. Inter-Reader Evaluation of Low keV Images and Contrast Phase

Regarding agreement between readers, no statistically significant differences were found. There was no significant difference between the two readers for any of the (calculated) keV levels in either the arterial or portal venous contrast phases (Table 15).

### 3.5. Dose Evaluation

**Figure 3:**
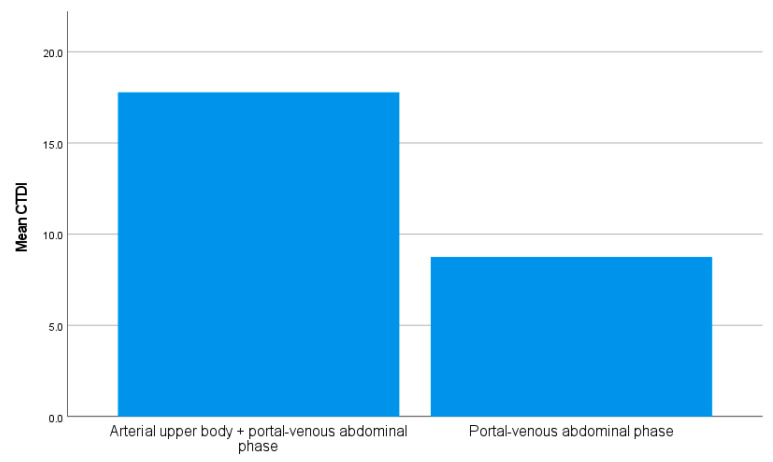
The mean computed tomography dose index (CTDI) can be reduced by 51% if the arterial contrast phase is omitted.

**Figure 4:**
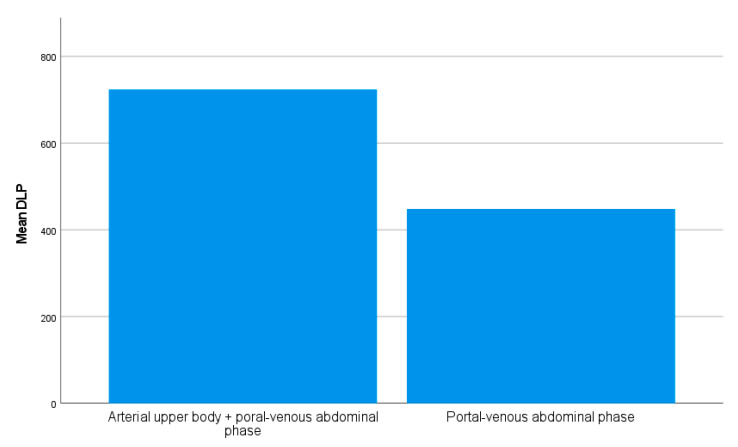
The mean dose-length product (DLP) can be reduced by 38% if the arterial contrast phase is omitted or even more if the pelvic region is not included in the examination area.

For PCD-CT, performing an additional arterial upper abdominal phase to the standard portal venous abdominal phase results in a 51% increase in the computed tomography dose index (CTDI) and a 38% increase in the dose-length product (DLP) (Table 16, Figure 3 and Figure 4).

## 4. Discussion

According to our results, the portal venous contrast phase, performed on a 1st generation PCD-CT in patients with hepatocellular carcinomas, yielded similar results with the arterial contrast phase; thus, holding potential for the reduction in radiation dose. Interestingly, the use of virtual monochromatic 40–70 keV images also did not really outperform the results of polychromatic T3D images, which potentially makes redundant the time-consuming post-processing of different sets of VMI. Hence, we found no significant difference in SNR between the arterial and portal venous phases, including between “T3D” and low keV images. Notably, the tumor-to-liver CNR was found to be significantly different between the arterial and portal venous contrast phases, but not between polychromatic “T3D” and low keV VMI. Expectedly, we found an increase in tumor-to-liver CNR, but also in SD in the arterial contrast phase at lower keV; whereas in the portal venous contrast phase, the tumor-to-liver CNR decreased at lower keV and concomitantly, the tumor-to-muscle CNR increased at lower keV in both arterial and portal venous contrast phases. This is in line with previously published results concerning image CNR [6]. There was no significant difference in SNR in the arterial contrast phase between T3D and low keV VMI, unlike in the portal venous contrast phase at 40 keV. The other keV levels yielded no significant difference over T3D.

Notably, the inter-reader agreement was good for most of the image readings. For this study, we used a standardized biphasic contrast-enhanced liver protocol where the applied energy as well as all other examinational parameters were kept comparable between the two phases. The arterial phase started at 30 s, whereas the portal venous phase started at 65 s. All liver lesions (100.0%) were detected in both the arterial phase and the portal venous phase by a mean lesion size of 4.2 ± 2.3 cm.

In this given clinical setting, the rationale for multiphasic contrast CT protocols is to improve the detection of hyper-vascular liver lesions; this topic has been intensively debated in the last two decades, advocating the role of an additional arterial contrast phase in patients presenting hyper-vascular tumors. Knowingly, the liver is supplied with blood from the portal vein to 70–80%; whereas, the bile ducts are supplied with blood from the hepatic artery. Most hepatic tumors are either exclusively or preponderantly supplied with arterial blood. Defining lesion conspicuity as the difference between lesion enhancement and parenchymal enhancement underlines the role of multiphasic studies. Based on this knowledge, timely separation of the dual blood supply would have the benefit of a higher lesion-to-liver ratio in the arterial phase, eventually coupled with a lower lesion-to-liver ratio in the portal venous phase. However, angiographic studies have shown that the portal venous phase already begins 5–6 s after contrast material injection in the celiac or splenic artery. This data suggest that with slower scanners, as used in the past, the arterial dominant phase already included some portal venous “contamination” affecting the results [24]. Frederick et al. suggest that the arterial phase is completed in 44 s, and that therefore, the detection of hyper-vascular lesions could be compromised if image acquisition lasts longer [24]. However, Winkler et al. could not find any additional liver metastases from malignant melanoma in their cohort comparing the arterial (40 s delay) with the portal venous contrast phase (70 s delay) [25]. These authors described a higher challenge in the detection of benign, primarily hepatic, lesions like adenoma and focal nodular hyperplasia; presumably, due to their greater histologic resemblance with the liver parenchyma. Mitsuzaki et al. reported improvement in the detection of smaller hepatocellular carcinomas by performing an additional arterial phase [26]. However, in their study, these authors reported a time-to-peak in the aorta of 36 s, and 90 s for the liver. With more rapid scanners, these delay times are expected to be significantly shorter, and the protocol used in our study comprised a shorter delay time for the portal venous phase (65 s) which corresponds to a late capillary/early portal venous enhancement phase. For hyper-vascular tumors, the lesion-to-liver ratio is expected to fall significantly from the arterial phase to the portal venous phase, eventually becoming negative in the latter.

In recent years, with the increasing use of dual-energy technology, the optimization of tissue contrast became possible by using virtual mono-contrast images obtained at low keV [27,28]. In a similar approach, the use of a frequency-selective nonlinear blending algorithm significantly improved tissue contrast [29]. The approval of a 1st generation photon-counting detector CT has given an additional impetus to assess its strengths and limits resulting from the more efficient exploitation of the spectral information. Eliminating electronic noise, the PCD-CT increases lesion delineation, and by means of reading virtual mono-contrast images at lower keV, also increases the tissue contrast that is supposed to be beneficial for lesion depiction. Hence, using technological advancement, requirements that are considered mandatory such as multiphasic contrast protocols could be disputed again.

At this point, the emerging CT technique called photon-counting detector CT could be used for the purpose of dose reduction by maintaining image quality. The main difference between a conventional energy-integrating detector CT and a PCD-CT is that the former uses indirect conversion technology, with a layer of scintillators converting X-ray photons into visible light which are consequently detected by a photodiode and converted into electronic signals, whereas the latter directedly converses X-ray photons into electron–hole pairs by using a semiconductor detector material with a better electron yield. Electronic noise is usually detected as a low-amplitude signal, and thus by setting the low-energy threshold to be slightly higher than the energy level associated with the electronic noise signal amplitude, electronic noise can be excluded readily from the measured count data.

Low-energy threshold images (referred to as T3D by the manufacturer) were developed as a surrogate of classical polychromatic 120 KVP images including photon energies from 20 keV to 120 keV. The way in which these polychromatic energies are weighed increases image contrast, which proved superior to classical 120 KVP; which again, seems to explain, in addition, the lesser impact of low keV images over T3D in our study compared to previous reports [11,30,31].

Our study has some limitations. First, it is just a preliminary study as our series does not have enough subjects for a vast statistical evaluation. Second, the standard of care, which in this case was MRI and CT follow-up, was performed within a time window that theoretically might have changed the number of lesions detectable by CT as compared to MRI. Third, this study does not intend to question the benefit of multiphasic imaging protocols generally. For many clinical questions, e.g., while dealing with the assessment of treatment response or for characterization of certain tumor entities, multiphasic protocols still have their justifications. Nevertheless, a reevaluation of established examinational protocols should be taken into consideration given that image quality is rapidly improving.

In summary, the additional arterial contrast phase showed a better contrast ratio for the lesion-to-background ratio compared to the portal venous contrast phase. Especially at 40 keV, the arterial phase showed the best results for the detection of hyper-arterialized HCC lesions. However, these objective findings were not confirmed by the subjective reading of the radiologists. In light of currently tighter regulations with respect to patient dose, these preliminary results should prompt larger studies aimed at the optimization of CT examinational protocols in the era of PCD-CT.

## Figures and Tables

**Table 1:** Patient characteristics.

	Patient Characteristics (*n* = 49)
Age (mean ± SD)	66.9 ± 11.2 years
Sex	8 females
Tumor entity	HCC (100%)
Previous exam (MRI)	94%
Size (cm, mean ± SD)	4.2 ± 2.3 cm
Weight (kg, mean ± SD)	74.1 ± 15.8 kg
BMI (mean ± SD)	24.3 ± 4.8

**Table 2:** The number of delineable lesions in the arterial and portal venous contrast phases, sorted by number.

Number of Lesions	art-Phase	pv-Phase	*p*-Value
1 lesion	19	19	>0.05
1–5 lesions	7	7	>0.05
5–10 lesions	5	5	>0.05
>10 lesions	18	18	>0.05

**Table 3:** SNR and CNR of low-energy threshold images (T3D). SNR: signal-to-noise ratio; CNR: contrast-to-noise ratio; art-phase: arterial upper abdomen phase; pv-phase: portal venous abdominal phase.

T3D	art-Phase	pv-Phase	*p*-Value
SNR	6.58 ± 2.86	5.93 ± 2.97	0.416
CNR liver-to-muscle	1.40 ± 0.42	1.73 ± 0.38	0.004
CNR tumor-to-liver	1.13 ± 0.49	0.79 ± 0.30	0.004
CNR tumor-to-muscle	1.53 ± 0.76	1.36 ± 0.60	0.366

**Table 4:** Comparison of SNR of arterial and portal venous phases.

SNR	art-Phase	pv-Phase	*p*-Value
T3D	6.58 ± 2.86	5.93 ± 2.97	0.208
70 keV	3.41 ± 1.59	3.21 ± 1.16	0.300
60 keV	4.61 ± 3.00	4.09 ± 1.90	0.222
50 keV	4.45 ± 2.38	5.61 ± 6.04	0.180
40 keV	5.00 ± 2.84	5.49 ± 4.59	0.139

**Table 5:** Comparison of CNR_tumor-to-liver_ of arterial and portal venous phases.

CNR_tumor-to-liver_	art-Phase	pv-Phase	*p*-Value
T3D	1.13 ± 0.49	0.79 ± 0.06	0.002
70 keV	1.15 ± 0.48	0.81 ± 0.30	0.002
60 keV	1.22 ± 0.59	0.78 ± 0.40	0.001
50 keV	1.34 ± 0.79	0.81 ± 0.44	0.002
40 keV	1.55 ± 0.98	0.80 ± 0.43	<0.001

**Table 6:** Image noise (standard deviation of the psoas muscle).

Image Noise	art-Phase	pv-Phase	*p*-Value
T3D	11.6 ± 4.22	11.8 ± 4.80	0.893
70 keV	24.4 ± 6.79	24.2 ± 6.56	0.926
60 keV	21.0 ± 5.72	21.1 ± 4.01	0.921
50 keV	25.0 ± 5.85	25.3 ± 5.54	0.888
40 keV	29.4 ± 6.26	30.2 ± 8.50	0.710

**Table 7:** Comparison of arterial SNR between T3D and low keV images.

SNR Art	T3D	70 keV	60 keV	50 keV	40 keV
T3D	x	<0.001	0.090	0.120	0.520
70 keV	<0.001	x	1.000	1.000	0.678
60 keV	0.091	1.000	x	1.000	1.000
50 keV	0.115	1.000	1.000	x	1.000
40 keV	0.520	0.678	1.000	1.000	x

**Table 8:** Comparison of portal venous SNR between T3D and low keV images.

SNR pv	T3D	70 keV	60 keV	50 keV	40 keV
T3D	x	0.006	0.389	1.000	1.000
70 keV	0.006	x	1.000	0.958	0.549
60 keV	0.389	1.000	x	1.000	1.000
50 keV	1.000	0.948	1.000	x	1.000
40 keV	1.000	0.549	1.000	1.000	x

**Table 9:** Comparison of arterial CNR_liver-to-muscle_ between T3D and low keV images.

CNR_liver-to-muscle_ Art	T3D	70 keV	60 keV	50 keV	40 keV
T3D	x	1.000	1.000	0.166	0.023
70 keV	1.000	x	1.000	0.323	0.053
60 keV	1.000	1.000	x	1.000	0.890
50 keV	0.166	0.323	1.000	x	1.000
40 keV	0.023	0.053	0.890	1.000	x

**Table 10:** Comparison of portal venous CNR_liver-to-muscle_ between T3D and low keV images.

CNR_liver-to-muscle_ pv	T3D	70 keV	60 keV	50 keV	40 keV
T3D	x	1.000	0.037	0.000	0.000
70 keV	1.000	x	0.633	0.003	0.000
60 keV	0.037	0.633	x	1.000	0.013
50 keV	0.000	0.003	1.000	x	1.000
40 keV	0.000	0.000	0.013	1.000	x

**Table 11:** Comparison of arterial CNR_tumor-to-liver_ between T3D and low keV images.

CNR_tumor-to-liver_ Art	T3D	70 keV	60 keV	50 keV	40 keV
T3D	x	0.853	0.543	0.235	0.054
70 keV	0.853	x	0.659	0.295	0.069
60 keV	0.543	0.659	x	0.513	0.143
50 keV	0.235	0.295	0.513	x	0.408
40 keV	0.054	0.069	0.143	0.408	x

**Table 12:** Comparison of portal venous CNR_tumor-to-liver_ between T3D and low keV images.

CNR_tumor-to-liver_ pv	T3D	70 keV	60 keV	50 keV	40 keV
T3D	x	0.800	0.962	0.801	0.903
70 keV	0.800	x	0.794	0.963	0.933
60 keV	0.962	0.794	x	0.791	0.881
50 keV	0.801	0.963	0.791	x	0.909
40 keV	0.903	0.933	0.881	0.909	x

**Table 13:** Comparison of arterial CNR_tumor-to-muscle_ between T3D and low keV images.

CNR_tumor-to-muscle_ art	T3D	70 keV	60 keV	50 keV	40 keV
T3D	x	1.000	1.000	0.633	0.076
70 keV	1.000	x	1.000	1.000	0.260
60 keV	1.000	1.000	x	1.000	1.000
50 keV	0.663	1.000	1.000	x	1.000
40 keV	0.076	0.260	1.000	1.000	x

**Table 14:** Comparison of portal venous CNR_tumor-to-muscle_ between T3D and low keV images.

CNR_tumor-to-muscle_ pv	T3D	70 keV	60 keV	50 keV	40 keV
T3D	x	0.383	0.159	0.031	0.003
70 keV	0.383	x	0.444	0.131	0.016
60 keV	0.159	0.444	x	0.534	0.117
50 keV	0.031	0.131	0.534	x	0.307
40 keV	0.003	0.016	0.117	0.307	x

**Table 15:** Data are presented as median qualitative image analysis scores; data in parentheses are interquartile ranges.

	40 keV			50 keV			60 keV			70 keV		
	art.	ven	*p*-Value	art.	ven.	*p*-Value	art.	ven.	*p*-Value	art.	ven.	*p*-Value
Image Quality												
Reader 1	3 (3 – 3)	3 (2 – 3)	0.712	3 (3 – 3)	3 (3 – 3)	0.731	4 (4 – 4)	4 (4 – 4)	1.000	4 (3 – 4)	4 (4 – 4)	0.698
Reader 2	3 (3 – 3)	3 (2 – 3)	0.702	3 (3 – 3)	3 (3 – 3)	0.878	4 (4 – 4)	4 (4 – 4)	1.000	4 (4 – 4)	4 (4 – 4)	0.995
Vessel depiction												
Reader 1	4 (3 – 4)	3 (3 – 3)	0.111	4 (3 – 4)	3 (3 – 4)	0.209	4 (4 – 4)	4 (3 – 4)	0.635	4 (4 – 4)	4 (4 – 4)	0.778
Reader 2	4 (4 – 4)	3 (3 – 3)	0.158	4 (4 – 4)	3 (3 – 4)	0.086	4 (4 – 4)	4 (3 – 4)	0.721	4 (4 – 4)	4 (4 – 4)	0.854
Lesion depiction												
Reader 1	3 (2 – 3)	3 (2 – 3)	0.892	3 (2 – 4)	3 (2 – 4)	0.878	3 (2 – 3)	3 (3 – 4)	0.807	4 (3 – 4)	4 (3 – 4)	0.552
Reader 2	3 (2 – 4)	3 (2 – 4)	0.924	3 (2 – 4)	3 (3 – 4)	0.652	3 (2 – 3)	3 (3 – 4)	0.733	4 (3 – 4)	4 (4 – 4)	0.103

**Table 16:** Art-phase: arterial upper abdomen phase; pv-phase: portal venous abdominal phase (including also the pelvic region); CTDI: computed tomography dose index; DLP: dose-length product; SD: standard deviation.

PCD-CT	art-Phase	pv-Phase	Added	Dose Savings (%)
CTDI (mean ± SD)	9.03 ± 3.59	8.75 ± 2.99	17.78 ± 6.40	51%
DLP (mean ± SD)	275 ± 133	448 ± 157	724 ± 272	38%

## Data Availability

Data is contained within the article.

## Abbreviations

CNR: contrast-to-noise ratio; studies: volumetric CT dose index; DLP: dose-length product; DSCT: dual-source CT; PCD-CT: photon-counting detector CT; SNR: signal-to-noise ratio.
